# Skeletal muscle dysfunction in amyotrophic lateral sclerosis: a mitochondrial perspective and therapeutic approaches

**DOI:** 10.1007/s10072-024-07508-6

**Published:** 2024-04-27

**Authors:** Gokhan Burcin Kubat, Pasquale Picone

**Affiliations:** 1 Department of Mitochondria and Cellular Research, Gulhane Health Sciences Institute, University of Health Sciences, Ankara, Turkey; 2 Istituto Per La Ricerca E L’Innovazione Biomedica, Consiglio Nazionale Delle Ricerche, Via U. La Malfa 153, 0146 Palermo, Italy

**Keywords:** Amyotrophic lateral sclerosis, Mitochondria, Skeletal muscle dysfunction, Mitochondrial transplantation

## Abstract

Amyotrophic lateral sclerosis (ALS) is a progressive and fatal neuromuscular disease that results in the loss of motor neurons and severe skeletal muscle atrophy. The etiology of ALS is linked to skeletal muscle, which can activate a retrograde signaling cascade that destroys motor neurons. This is why satellite cells and mitochondria play a crucial role in the health and performance of skeletal muscles. This review presents current knowledge on the involvement of mitochondrial dysfunction, skeletal muscle atrophy, muscle satellite cells, and neuromuscular junction (NMJ) in ALS. It also discusses current therapeutic strategies, including exercise, drugs, stem cells, gene therapy, and the prospective use of mitochondrial transplantation as a viable therapeutic strategy.

## Introduction

Amyotrophic lateral sclerosis (ALS) is the most common motor neuron disease in adults and is characterized by the degeneration of motor neurons (MN) in the nervous system [1]. Skeletal muscle dysfunction may contribute to the development of muscle atrophy, degeneration of neuromuscular connections, and ultimately loss of motor neurons in ALS patients [2]. Skeletal muscle in ALS is primarily affected by oxidative stress, mitochondrial dysfunction, and bioenergetic abnormalities [3]. The brain is an organ that consumes a high amount of energy and produces ATP molecules through oxidative phosphorylation in the mitochondria [4]. The reason for this is that neurons have high metabolic needs and mitochondria have a crucial role in the fulfilment of these needs [5]. Skeletal muscle atrophy has been implicated in ALS and is associated with reduced life expectancy [6]. Currently, only four drugs are approved for treating ALS due to their limited impact on disease progression: riluzole, edaravone, sodium phenylbutyrate/taurusodiol, and tofersen [7, 8]. Exercise can be a beneficial therapeutic strategy to improve the overall muscle health of ALS patients [9]. Stem cell treatment and gene therapies offer potential approaches to directly address the loss of MNs through several possible mechanisms in ALS [10, 11]. Mitochondrial transplantation has been suggested as a potential treatment for neurodegenerative diseases such as Parkinson’s, Alzheimer’s, and ALS based on promising preclinical studies [12]. This review summarizes the significance of mitochondrial dysfunction, skeletal muscle atrophy, muscle satellite cells, and neuromuscular junction (NMJ) in ALS. Additionally, current therapeutic approaches, including exercise, drugs, stem cells, gene therapy, and the potential use of mitochondrial transplantation as a therapeutic strategy, are reviewed.

## Amyotrophic lateral sclerosis

ALS is a degenerative disease of the central nervous system that is fatal. It is characterized by the progressive death of motor neurons in the brain and spinal cord. Currently, there is no known cure for ALS and its causes remain unknown. The incidence of ALS typically occurs in individuals between the ages of 60 and 79, although it can vary based on ancestral background and increases with age [13]. In Europe and North America, the incidence ranges from 1.71 to 1.89 per 100,000 [13]. ALS is characterized by a dysfunction of the motor neurons (MN) which affects various structures including the bulbar, cervical, thoracic, and lumbar segments. This dysfunction leads to a progressive deterioration of the skeletal muscles involved in limb movement, resulting in dysphagia, dysarthria, and breathing difficulties [13, 14]. The average survival time from diagnosis for ALS patients is 2–3 years. Only 25% of patients survive for 5 years, and 5–10% survive for 10 years from diagnosis. Approximately 10% of ALS cases are hereditary (familial ALS), while the remaining 90% are sporadic [13, 14]. It is a multifactorial disease in which various genes and pathophysiological processes contribute to the condition. Understanding this heterogeneity will be essential in finding effective treatments.

## Mitochondrial dysfunction in ALS

**Fig. 1:**
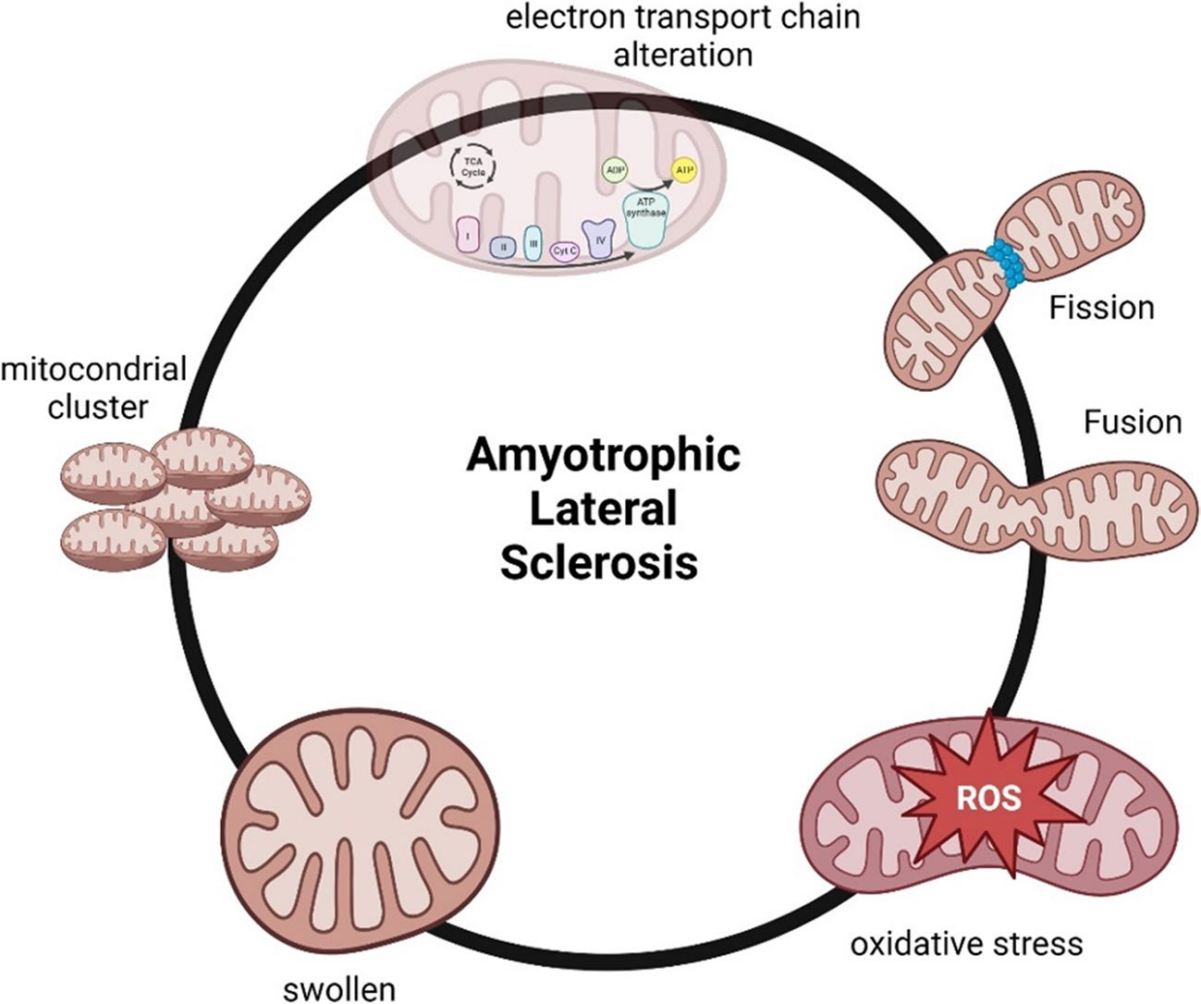
Mitochondrial dysfunction in ALS

Mitochondria are organelles that are essential for several cellular processes, including energy production, calcium storage, lipid production, and cell death. The brain, which represents only 2% of the body mass, uses 20% of the ATP produced by the body at rest [15]. Mitochondria are particularly important in neurons because of their high metabolic demands [5]. It is therefore unsurprising that numerous neurodegenerative diseases are characterized by mitochondrial dysfunction, including ALS (Fig. 1).

In 1993, the revolutionary discovery was made that the protein superoxide dismutase 1 (SOD1), which is produced by the ALS1 gene, was the cause of familial ALS (fALS) [16]. SOD1 is a superoxide scavenger that localizes in the cytosol and mitochondria [17]. This discovery highlighted how alterations in redox conditions are an important factor in this disease [18]. Alterations in mitochondrial redox status, structure, dynamics, and functionality have been widely reported in ALS patients and model systems. There is strong evidence to suggest that mitochondria play a significant role in the pathogenesis of the disease. Approximately 2% of ALS cases are caused by mutations in SOD1 [19, 20]. In addition to SOD1, mutations in over 40 genes, such as TAR DNA binding protein (TARDBP; TDP-43), fused in sarcoma (FUS), and C9orf72, are also associated with ALS [19–21]. Many of the identified genes associated with ALS play a role in mitochondrial and mitochondrial-associated functions [21].

In the context of ALS, oxidative stress, derived from mutant SOD1 leading to high concentrations of reactive oxygen species (ROS), also affects the metabolic functions of mitochondria-ER contact communication leading to changes in well-known pathologies such as endoplasmic reticulum (ER) stress, inflammation, and motor neuron death [22]. Increased mitochondrial Ca^2+^ levels have also been reported in G93A Cu/Zn superoxide dismutase–mutant mice, further supporting the critical role of mitochondrial dysfunction in the pathogenesis of ALS [23]. Research increasingly supports the theory that mitochondrial dysfunction plays an active role in the development of ALS.

### Mitochondrial structural alteration

Structural damage to mitochondria has been reported to occur in the early stages of the disease, indicating that mitochondrial alteration is a source of degeneration rather than a consequence [24] (Fig. 1). In ALS patients, one of the initial observations is the structural alteration and aggregation of mitochondria in motor neurons and the mitochondria appear swollen and vacuolated [25, 26]. In animal and cellular models of ALS, mitochondria undergo constant morphological changes, tending towards fragmentation and the formation of abnormal clusters along the axon [27]. The discovery of CHCHD10 mutation protein, which is localized in the contact sites between the inner and outer membranes of the mitochondria, has demonstrated that alterations in mitochondrial structure can contribute to the etiology of ALS [28].

### Oxidative stress

SOD1 is associated with both mitochondrial dysfunction and oxidative stress [29] (Fig. 1). Pickles et al. reported that SOD1G93A rats and SOD1G37R mice were more susceptible to oxidative stress and impaired mitochondrial function due to the accumulation of misfolded SOD1 in spinal cord mitochondria [30]. Oxidative stress can contribute to the development of sporadic ALS, while familial ALS is attributed to mutations in the enzymes Cu, Zn, and SOD1 [31]. Mutations in SOD1, TDP-43, and C9orf72, which are associated with familial amyotrophic lateral sclerosis (fALS), can lead to mitochondrial dysfunction via increased ROS [32]. Loss-of-function mutations in the FUS gene have been shown to cause DNA strand breaks, which increase sensitivity to oxidative stress. This indicates that normal protein activity guards against oxidative stress in ALS [33]. Increased levels of ROS allow greater accumulation of glutamate in the synapse, leading to increased excitation of glutamate receptors. This, in turn, results in an increase in calcium influx into motor neurons and mitochondria, ultimately causing mitochondrial damage [34]. Furthermore, the mitochondria in the spinal cord of ALS patients showed a decrease in the activity of oxidative phosphorylation complexes I + III, II + III, and IV, resulting in reduced respiration and ATP synthesis [32].

In ALS, there is a significant correlation between the activation of Caspase 3 and an increase in protease activity. The disruption of Caspase 3 resulted in the production of abnormal TDP-43/mitochondrial protein interactions. Inhibiting Caspase 3 in both murine and human skeletal muscle cells resulted in the formation of TDP-43 aggregates and impaired mitochondrial activity [35]. Mitophagy is linked to central neurodegenerative disorders and oxidative stress conditions. ALS patients may have genetic mutations in genes that regulate mitophagy, such as optineurin and p62/sequestrosome-1 [36]. Magrì et al. observed that impaired mitophagy activation may be linked to the overactivation of the ERK1/2 pathway, as evidenced by a decrease in expression of the mitophagy marker Atg12 and increased levels of TSPO [37].

### ALS and mitochondrial dynamics

Mitochondria are highly dynamic organelles that undergo continuous processes such as trafficking, fission, fusion, and turnover, which are fundamental for maintaining mitochondrial activities (Fig. 1). The processes of mitochondrial fission and fusion are rigorous regulated by several dynamin-related GTPases. Mitochondrial fission (division of a single organelle into two or more independent structures) in mammals involves at least dynamin-like protein 1 (DRP1) and its mitochondria recruitment factors such as Fis1, Mff, MiD49, and MiD51 [38]. On the other hand, mitochondrial fusion (opposite reaction to fission) is regulated by three large GTPase proteins: mitofusin 1 (Mfn1), mitofusin 2 (Mfn2), and optic atrophy protein 1 (OPA1) [39]. The morphology of cellular mitochondria is regulated by a continuous occurrence of these actions and their balance. Abnormalities in mitochondrial dynamics have been identified in ALS, suggesting that alterations in mitochondrial dynamics may underlie the pathological mechanisms of ALS. A recent study in SOD1 G93A transgenic mice found that levels of fission protein and fusion regulators (DRP1, Fis1, Mfn1, and OPA1) increase before disease onset, consistent with changes in mitochondrial morphology [40]. Additionally, changes in the expression of DRP1 and Mfn1 have also been observed in the spinal cord of transgenic mice that overexpress wild-type TDP-43 [41]. These results on the alteration of mitochondrial fission/fusion highlight that mitochondrial dynamics could probably be a process involved in mitochondrial dysfunction in ALS.

## Skeletal muscle impairments

### Skeletal muscle atrophy

**Fig. 2:**
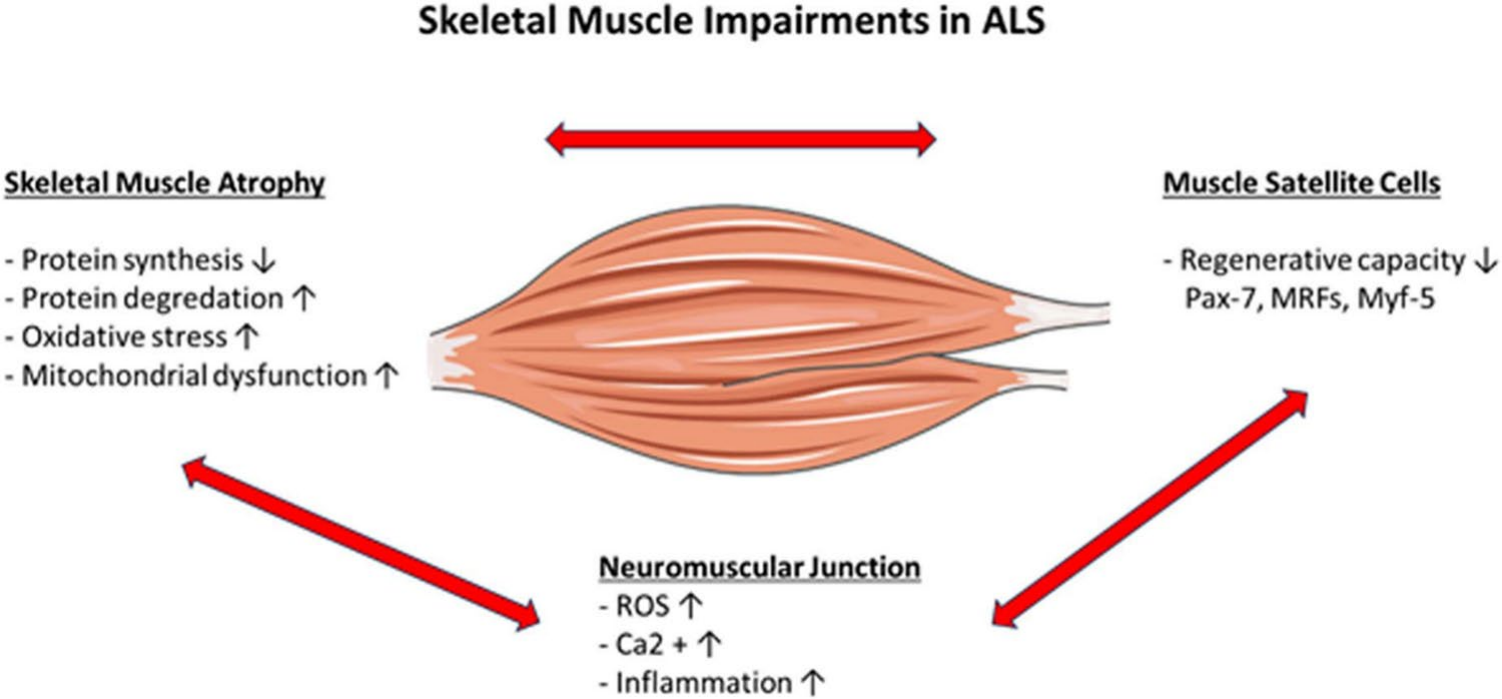
Skeletal muscle impairments in ALS

Skeletal muscle and motor neurons operate as a unified functional entity, mutually affecting each other. In the context of ALS, the term “amyotrophic” refers to the muscle atrophy and weakness observed in lower motor neuron disorders [42] (Fig. 2). As damage to the skeletal muscle may accelerate MN loss in ALS, retrograde neurodegeneration of MNs may play a significant role in the pathogenesis of the disease [43]. Studies have shown that rapid weight loss in ALS patients is associated with a shorter life expectancy. Additionally, severe skeletal muscle atrophy has been found to have a negative impact on the prognosis of various diseases [6]. Patients with ALS have a distinct muscle pattern characterized by progressive atrophy and wasting. In addition, some mechanisms have been identified that are common to other neuromuscular diseases, such as sarcopenia [44].

One study revealed pathological changes consistent with denervation, reinnervation, and myopathy in quadriceps muscle tissue samples collected from 24 patients diagnosed with ALS. Furthermore, the morphometric data showed a correlation between the duration and average diameter of type I fibers and the duration and hypertrophic factor of type II fibers [45]. The carboxyl-terminal modulator protein (CTMP) influences Akt signaling and skeletal muscle physiology. In hindlimb skeletal muscle, CTMP protein expression increased significantly during the late stages of ALS. Indicators of autophagy, lysosome formation, and atrophy-related cellular degradation processes were significantly increased, corresponding to higher CTMP expression and reduced Akt phosphorylation [46]. Atrogin-1 expression increased in human samples of ALS, while Murf-1 expression remained unchanged [47]. The expression of SOD1-G93A results in a reduction of the Akt/phosphatidylinositol 3-kinase pathway, which is associated with the activation of forkhead box O3, leading to skeletal muscle atrophy in ALS [48]. As ALS progresses, there is a strong correlation with the expression of histone deacetylase 4 (HDAC4) in the skeletal muscle. Moreover, reducing HDAC4 in SOD1 mice led to a deterioration in muscle function and accelerated muscle wasting [49]. Peroxisome proliferator-activated receptor co-activator-1 (PGC-1) is a transcriptional co-activator that is important for mitochondrial biogenesis and its levels decrease with skeletal muscle atrophy. This statement suggests that PGC-1 may have the ability to regulate skeletal muscle mitochondria in ALS on a molecular level [50]. Muscle specimens from patients with FUS mutations showed skeletal muscle atrophy and a decrease in FUS subsynaptic concentration [51]. In ALS-FUS patients, cytoplasmic FUS was observed in the skeletal muscle near a small number of mitochondria. This colocalization was associated with mitochondrial swelling, disordered cristae, and downregulation of mitochondrial oxidative phosphorylation complexes [52]. It is currently unclear whether the skeletal muscle atrophy observed in ALS is solely due to denervation or if it also contributes to a weakening of internal muscle processes. The skeletal muscles of ALS patients exhibit malfunctions in cell metabolism, protein and protein aggregate removal and degradation, RNA activities, and muscular atrophy pathways.

### Muscle satellite cells

During homeostasis, muscle satellite cells (SCs) are quiescent in mature tissues and are stimulated in response to acute muscle injury or chronic degenerative disease [53]. Normally, SCs remain inactive but respond to stimuli such as acute injury, muscular denervation, or exercise. In response to stimulation, SCs proliferate, differentiate into myoblasts, and interact with myofibers to aid in the repair of muscle fibers. During the mitotic phase, quiescent SCs express members of the myogenic regulatory factor (MRF) family, including the paired box 7 transcription factor (Pax7) and myogenic transcription factor-5 (Myf5) [54].

Furthermore, myoblast cells derived from patients with ALS exhibited higher levels of MyoD mRNA than control cultures, while demonstrating equivalent levels of Pax7 mRNA, indicating a more committed state [55]. ALS is associated with a gradual deterioration of NMJ integrity, which is linked to changes in the function and quantity of SCs [56, 57] (Fig. 2). ALS is characterized by the degeneration of motor neurons and the reduction of neuromuscular junctions. These changes are caused by imbalanced proteostasis and an inadequate unfolded protein response (UPR) [58, 59]. The regeneration and growth of the skeletal muscle are substantially impacted by the activation of various myogenic phases and the quantity of stem cells [60]. A study has reported that the activation of SCs and regenerating fibers was among the various degrees of denervation- and reinnervation-related changes in muscle samples affected by ALS. Although SCs taken from ALS patients were able to multiply in vitro, they displayed an abnormal senescent-like appearance, as evidenced by the upregulation of senescence markers such as senescence-associated (SA)-galactosidase activity and p16 expression [61]. During the regeneration of the NMJ, SCs become activated and proliferate [54]. Deficiency in SCs results in muscle fiber shrinkage, increased connective tissue between myofibers, and reduced myofiber/NMJ connection [54]. The reduced myogenic capacity in muscle cultures and tissues has only been elucidated in a few ALS patients. However, further research is needed to confirm and extend this understanding.

### Neuromuscular junction

The NMJ is where muscle and nerve cells interact. It consists of the postsynaptic muscle membrane, the synaptic cleft enclosed by a basal lamina, and the presynaptic region, which includes the nerve terminal [62]. NMJ degradation is considered a significant early indicator of motor neuron loss in ALS [63] (Fig. 2). Both mitochondrial dysfunction and NMJ dysfunction hinder axonal transport, which is caused by an interruption in presynaptic action potential generation [64]. Abnormalities in muscle mitochondria were observed only in the final stages of the disease, indicating degeneration of MNs in the spinal cord [65].

There is a connection between the functioning of the NMJ and genes associated with ALS, including CHMP2B. The CHMP2B mutation affects the structure of presynaptic terminals and synaptic transmission, resulting in a shift from type II muscle fibers to more type I fibers [66]. The symptoms of ALS linked to overexpression of the human mutant TDP43 include progressive motor impairment, muscle wasting, compromised NMJ integrity, and degeneration of MNs [67]. SOD1- and TDP-43-mutant mice show abnormal mitochondrial morphology, which may trigger a cascading response that leads to the degeneration of motor neurons [27]. Mutant SOD1 causes the degradation of mitochondrial Rho GTPase 1 (Miro1), hindering the transit of mitochondrial axons [68]. Miro1 promotes the attachment of mitochondria to kinesin 1 by impacting the levels of cytosolic Ca^2+^ [69]. MN degeneration can occur due to NMJ disruption caused by mitochondrial dysfunction and energy depletion in skeletal muscles affected by ALS.

## Therapeutic approaches for ALS

### Exercise

**Fig. 3:**
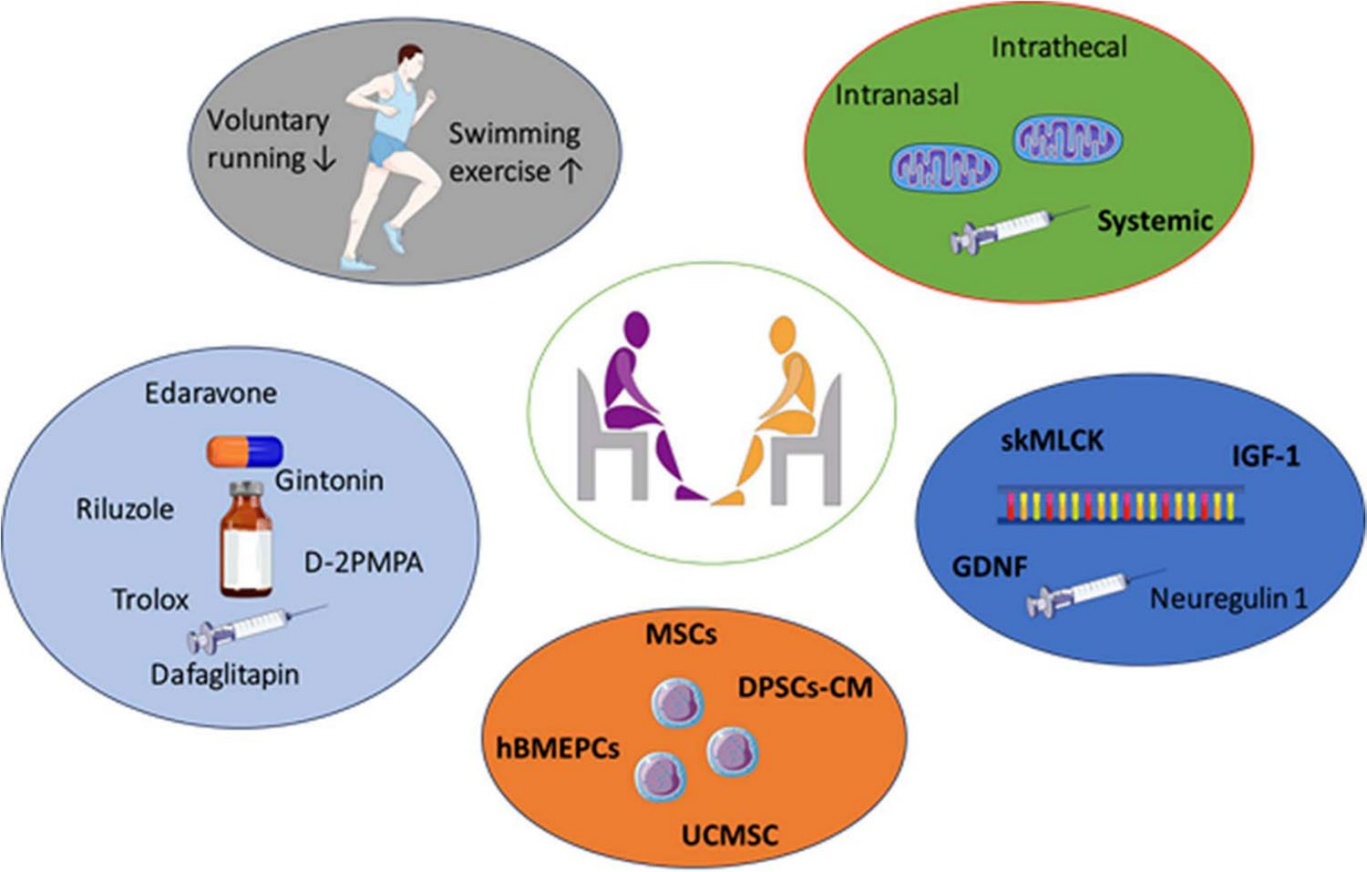
Therapeutic approaches for ALS

Exercise is often used as an alternative to therapy and has the potential to improve overall muscular strength and cardiovascular health in people with ALS [9, 70] (Fig. 3). This could improve the quality of life for ALS patients [71]. A study found that swimming may relieve muscle atrophy in ALS mice by restoring FOXO3a signaling and significantly reducing the loss of skeletal muscle mass [72]. A mechanism related to autophagy has been shown to be involved in the recovery of the ALS-sensitive tibialis muscle following swimming exercise [73]. ALS patients can benefit from exercise or adeno-associated virus treatment, which has been shown to improve motor function and increase survival rates [74]. Regular physical activity does not have a negative impact on the onset of familial ALS in transgenic mice. Additionally, it provides direct neuroprotective benefits to the degenerating neurons [75]. Although some studies suggest that exercise may be beneficial for ALS patients, others indicate that it may accelerate the progression of the disease. Mild-to-moderate endurance exercise is recommended as a beneficial therapy, while intense endurance exercise appears to have no impact or to be detrimental in ALS [76]. Mahoney et al. observed that frequent, intense endurance exercise exacerbated the motor function loss and decreased survival in male SOD1G93A mice, but not females [77]. Carreras et al. found that high-intensity endurance exercise substantially accelerated the development of motor function impairments in SOD1G93A mice [78]. Repetitive voluntary running has been found to impede neuromuscular transmission, worsen neuromuscular decline, and amplify muscle atrophy, ultimately exacerbating the course of the disease [79]. Julian et al. found an association between daily physical activity and increased ALS risk, and the primary risk factor for ALS appears to be rapid anaerobic exercise or intense physical activity [80]. Further research is needed to establish the most effective exercise regimens and dosing guidelines for people diagnosed with ALS.

### Drugs

The drugs riluzole, edaravone, sodium phenylbutyrate/taurursodiol, and tofersen are currently approved by the US Food and Drug Administration (FDA) for the treatment of ALS (Fig. 3). Riluzole is the only drug administered therapeutically as an inhibitor of glutamate release among ALS patients [42]. It inhibits the reuptake of glutamate in synapses of motor neurons and deactivates voltage-dependent sodium channels [81]. A daily dose of 100 mg was associated with a 35% decrease in mortality, which extended survival by almost 3 months over the course of a year [81]. Edaravone has been shown to have cytoprotective effects by preventing peroxidation and acting as a ROS scavenger, delaying the onset of ALS, and protecting neurons by reducing ROS [82]. The combination of sodium phenylbutyrate and taurursodiol in fixed doses is believed to reduce endoplasmic reticulum stress and mitochondrial malfunction, thereby decreasing neuronal cell death [83]. The FDA approved tofersen, a new antisense oligonucleotide (ASO) medication in April 2023 for the treatment of ALS [8]. The production of the SOD1 protein is essentially inhibited by tofersen, which binds to and promotes the degradation of SOD1 mRNA derived from mutant SOD1 genes [84]. Despite the continued prevalence of their usage, new medications are being investigated for the treatment of ALS.

Masitinib (NCT03127267) is a tyrosine kinase inhibitor that targets macrophages, mast cells, and microglia cells to reduce the neuroinflammatory activity associated with ALS [85]. Researchers hypothesize that it affects the central and peripheral nerve systems via modulating immunological responses in several different regions, including mast cells, macrophages, and microglia [8]. Honokiol improved mitochondrial efficiency and morphology by enhancing mitochondrial dynamics in SOD1G93A cells. As a result, SOD1G93A transgenic mice survived longer and showed improved motor abilities [86]. The skeletal muscle of SOD1G93A mice shows a significant increase in ALCAT1 protein expression. Dafaglitapin, an ALCAT1 inhibitor, has been demonstrated to decrease the accumulation of SOD1G93A protein and mitochondrial dysfunction, thereby reducing motor neuron failure, neuronal inflammation, and skeletal muscle atrophy [87]. Administration of IL-10 in the hindlimb skeletal muscles improved the motor ability of mice with ALS by increasing the pro-regenerative action of muscle satellite cells and macrophages. As a result, muscle atrophy and motor neuron loss in ALS were delayed [88]. In SOD1G93A mice, systemic administration of D-2PMPA resulted in the absorption of D-2PMPA in muscle macrophages. This treatment significantly increased grip strength and prevented degradation of neuromuscular junction innervation in the gastrocnemius muscles [89]. A herbal infusion containing ingredients such as *Glycyrrhiza uralensis* and *Atractylodes* was found to improve motor function and reduce MN loss, inflammation, and oxidative stress in SOD1G93A mice and to effectively regulate autophagy activity [90]. Gintonin improved the survival of MNs, reduced motor dysfunctions, decreased ferritin-related oxidative stress, and induced the production of brain-derived neurotrophic factors. Furthermore, it reduced immunoreactivity to ionized calcium-binding adapter molecule 1, S100, and Olig2 in ALS mice [91]. In rats with SOD1G93A, the antioxidant Trolox effectively restored mitochondrial function, preserving the structure of the neuromuscular junction in ALS [92]. There was a significant increase in survival in SOD1G93A mice treated with CoQ10 to reduce oxidative stress and improve mitochondrial function [93]. In addition, mitochondria-targeting drugs such as olesoxime (NCT01285583), nortriptyline, and cyclosporine have been shown to have neuroprotective effects in ALS cells and mouse models [94–96].

### Gene therapy

Although there is currently no effective treatment for ALS, gene therapy has the potential to lead to the discovery of new drugs (Fig. 3). Lentiviral vectors (LVs) were employed to test the RNAi method of treating fALS, and Raoul et al. reported that bilateral intraspinal injection of a VSV-G LV causing RNAi-mediated suppression of SOD1 delayed disease development and prevented MN loss in SOD1G93A mice [97]. The injection of adeno-associated virus serotype 6 (AAV6/skMLCK) encoding skeletal muscle–specific myosin regulatory light chain kinase (skMLCK) has been shown to enhance muscle function. In SOD1G37R mice, both twitch and tetanic force were increased [11]. The expression of the insulin-like growth factor (IGF-1) isoform preserved muscle quality and increased the function of stem cells in SOD1G93A mice. This expression induced regenerative pathways mediated by calcineurin, maintained neuromuscular junctions, and ultimately resulted in enhanced motor neuron survival [98]. In mice with ALS, gene therapy that involved neuregulin 1 reinstated motor function in hindlimb muscles and preserved motor neurons and innervated neuromuscular junctions while reducing glial reactivity [99]. After intravenous injection of adeno-associated vectors coding for glial cell line-derived neurotrophic factor (GDNF), the number of innervated NMJs increased, the survival of spinal MNs was prolonged, and glial reactivity was decreased in SOD1G93A mice [100]. Gene therapy has been successful in treating other motor disorders, such as SMA. Currently, clinical trials are underway for ASO-based gene therapies, while AAV-based treatments are still being investigated.

### Stem cells

Stem cells have the capacity for regeneration and are undifferentiated. Stem cell therapy shows promise as a strategy to directly address the loss of motor neurons through several potential pathways in ALS [10] (Fig. 3). In ALS mice, intravenously administered CD34 + cells derived from human bone marrow (hBM34 +) or endothelial progenitor cells (hBMEPCs) have been shown to prolong disease progression by restoring the blood-spinal cord barrier (BSCB) [101]. However, transferred hBMEPCs have been observed to be more effective than hBM34 + cells in reducing disease-related behavioral effects and significantly increasing the lifespan of mice with ALS [101]. The lifespan of transgenic SOD1G93A mice was extended through systemic administration of umbilical cord MSCs (UCMSC). Additionally, UCMSC reduced iNOS production and secretion of proinflammatory cytokines in the spinal cord. This ultimately inhibited microglia stimulation and astrogliosis, resulting in reduced inflammation [102]. The study found that human dental pulp stem cells (DPSCs-CM) prevented the death of motor neurons (MNs) caused by a deficiency in trophic factors and promoted the development of axons in ALS mice [103]. The combined intra-spinal and systemic injection of MSCs significantly impacted motor activity, grip strength, and lifespan in SOD1G93A rats. Furthermore, the treatment resulted in a greater number of larger motor neurons with lower rates of apoptosis [104]. Cell-to-cell communication is modified by trophic factors and extracellular vesicles (EVs) released by MSCs [105]. These free-cell materials could shield degenerating MNs and provide a potential cure for ALS. A research study highlighted the neuronal development of MSC exosomes and the presence of transcripts for various anti-inflammatory and antioxidant genes in ALS (SOD1G93A transgenic) primary motor neurons [106]. The available literature on stem cell therapy suggests that it is very promising. However, further carefully designed clinical trials are needed to properly confirm its efficacy.

### Mitochondrial transplantation

Mitochondrial transplantation (MT) has recently been applied to several diseases, including cardiovascular, musculoskeletal, liver, kidney, and neural disorders [107–109] (Fig. 3). As progressive mitochondrial damage leads to energy metabolism dysfunction, ATP production is reduced, ROS stress is increased, and calcium buffering is reduced, contributing to neuronal loss, a hallmark of both acute and chronic degenerative neurological disorders [110]. In the ischemic brain, MT improved motor function by reducing the effects of reperfusion/ischemia and decreasing infarct size [111]. Exogenously administered mitochondria prevented the growth of microglia and astrocytes, the generation of (ROS, and the deterioration of neurons in the hippocampus [112]. In cerebral ischemia, MT increased cell survival, decreased ROS and apoptosis, decreased infarct size, and ameliorated neurobehavioral deficits [113]. Transplanted mitochondria reduced DRP-1 protein production, demyelination, cell death, and inflammation, while improving mobility and sensory function [114]. Transplanting healthy mitochondria improved the deterioration of oligodendrocyte function, enhanced olig2 and lipid metabolism signaling, and restored locomotion in the ischemic brain [115]. In traumatic brain injury (TBI), the introduction of isolated mitochondria into neuronal cells led to a reduction in apoptosis and microglial activation, while improving sensory abilities [116]. Moreover, MT has been proposed as a potential strategy for preventing axonal degeneration in multiple sclerosis [117]. While further research is needed to improve delivery methods, MT is expected to alleviate the effects of neurodegenerative injury or disease [12, 118]. In this context, vesicles or hydrogels have recently been proposed to improve mitochondrial transfer [119, 120].

As stated in this review, there is considerable data on the role of mitochondria in ALS deterioration. However, there have been no studies on MT and ALS to date. Therefore, we hypothesize that increasing the number of viable mitochondria or their activity may benefit ALS patients. This hypothesis would require support from in vitro and in vivo studies.

## Conclusion

A mechanism associated with both the early and late phases of ALS has been identified as mitochondrial dysfunction. The presence of impairments in dynamics, oxidative phosphorylation, and mitochondrial oxidative stress suggests that this organelle could be a possible target for ALS treatment. In this review, we highlight how different approaches such as physical exercise, pharmacological treatments, gene therapy, and stem cell therapy individually or concomitantly could be a potential strategy to arrest mitochondrial dysfunction in ALS. Finally, we offer mitochondrial transplantation as a novel and possible therapy strategy for ALS.

## Data Availability

Data sharing not applicable to this article as no datasets were generated or analyzed during the current study.
